# Antiviral Targeting of Varicella Zoster Virus Replication and Neuronal Reactivation Using CRISPR/Cas9 Cleavage of the Duplicated Open Reading Frames 62/71

**DOI:** 10.3390/v14020378

**Published:** 2022-02-12

**Authors:** Betty W. Wu, Michael B. Yee, Ronald S. Goldstein, Paul R. Kinchington

**Affiliations:** 1 Graduate Program in Microbiology and Immunology, School of Medicine, University of Pittsburgh, Pittsburgh, PA 15213, USA; bww20@pitt.edu; 2 Department of Ophthalmology, School of Medicine, University of Pittsburgh, Pittsburgh, PA 15213, USA; michaelyee80@gmail.com; 3 Faculty of Life Sciences, Bar-Ilan University, Ramat Gan 5900002, Israel; ron.goldstein@biu.ac.il

**Keywords:** varicella zoster virus, latency, reactivation, genome cleavage, CRISPR/Cas9, AAV, antiviral therapies

## Abstract

Varicella Zoster Virus (VZV) causes Herpes Zoster (HZ), a common debilitating and complicated disease affecting up to a third of unvaccinated populations. Novel antiviral treatments for VZV reactivation and HZ are still in need. Here, we evaluated the potential of targeting the replicating and reactivating VZV genome using Clustered Regularly Interspaced Short Palindromic Repeat-Cas9 nucleases (CRISPR/Cas9) delivered by adeno-associated virus (AAV) vectors. After AAV serotype and guide RNA (gRNA) optimization, we report that a single treatment with AAV2-expressing *Staphylococcus aureus* CRISPR/Cas9 (saCas9) with gRNA to the duplicated and essential VZV genes ORF62/71 (AAV2-62gRsaCas9) greatly reduced VZV progeny yield and cell-to-cell spread in representative epithelial cells and in lytically infected human embryonic stem cell (hESC)-derived neurons. In contrast, AAV2-62gRsaCas9 did not reduce the replication of a recombinant virus mutated in the ORF62 targeted sequence, establishing that antiviral effects were a consequence of VZV-genome targeting. Delivery to latently infected and reactivation-induced neuron cultures also greatly reduced infectious-virus production. These results demonstrate the potential of AAV-delivered genome editors to limit VZV productive replication in epithelial cells, infected human neurons, and upon reactivation. The approach could be developed into a strategy for the treatment of VZV disease and virus spread in HZ.

## 1. Introduction

Varicella-zoster virus (VZV) is the human alphaherpesvirus that causes varicella (chickenpox) during primary infection and herpes zoster (HZ; commonly called “shingles”) when the virus reactivates from the latent state, often decades after the initial infection [1]. Without immune boosting through the use of HZ vaccines, it is estimated that one-third of the population will develop HZ in their lifetimes, with incidence rising with age and declining immune status caused by natural senescence, disease, or iatrogenic causes. HZ remains a public health concern because it is often complicated by scarring, bacterial infections, and acute pain that can be debilitating. A significant fraction of HZ patients develop difficult-to-treat chronic pain states termed post-herpetic neuralgia (PHN), which can be so severe that they reduce quality-of-life. HZ may also be followed by neurological, gastrointestinal, and vascular diseases [2,3,4], as well as potentially blinding complications that develop after facial zoster [5].

HZ incidence and severity are reduced by boosting the existing VZV-specific immunity using vaccines. The first HZ licensed vaccine (used since 2005) was based on a higher dose version of the live-attenuated VZV strain used in the varicella vaccine, and it reduced HZ incidence by half and the disease burden by two-thirds [6]. However, it was contra-indicated in immunocompromised patients who could develop vaccine virus-induced disease [7,8]. A more recent Federal Drug Administration (FDA)-approved vaccine is based on the novel AS01B adjuvant and purified VZV glycoprotein E (gE). This subunit vaccine has higher efficacy against HZ, but requires two doses, has frequent side effects or injection-site reactions and is not used worldwide [9,10,11]. Hence, the uptake of the HZ vaccines remains low. HZ disease can respond to antiviral treatment if it is initiated early, but current antivirals only target active viral replication. The latent state remains refractory to any known antiviral treatment [5,12,13]. Thus, VZV diseases still impact millions of people worldwide and there is a need for improved HZ treatments/prevention.

One of the main hurdles in developing novel VZV therapeutic strategies has been the difficulty in modeling VZV pathogenesis in animal models. VZV demonstrates high human specificity, and does not fully replicate or cause disease in rodents, compared to the modeling of infections and diseases caused by the related herpes simplex virus type 1 (HSV-1) [14]. Indeed, there is no in vivo immunocompetent model of human VZV-induced primary disease, a VZV latent state that is reactivatable, or HZ-like disease states resulting from reactivation [1,15]. Attempts to experimentally reactivate VZV from latently infected human cadaver ganglia have also not yet been successful [16,17]. VZV does replicate in fetal human tissues harbored in severe compromised immunodeficient (SCID) mice, which can be used to evaluate pathogenesis and antiviral studies [18,19,20], but their use can be cost-prohibitive and requires special animal-experimentation permissions. However, neuronal culture models have been developed that harbor latent VZV that can be experimentally reactivated. We previously reported neuronal cultures derived from human embryonic stem cells (hESC) that model VZV neuronal lytic replication, axonal transport, neuron-to-neuron spread, and a prolonged viral persistent state that could be experimentally reactivated by the interruption of the NGF signaling or alteration of histone/chromatin architecture [21,22,23]. These and similar neuronal models used by other groups [24,25,26,27,28,29,30] have now established the means to probe the VZV latent state and investigate the potential targeting of latent and lytic replicating genomes using gene editing. Targeted CRISPR/Cas9 and specific homing meganucleases have been explored to target HSV-infected neurons and in murine neuronal models of disease and latency in vivo [31,32,33], as well as human cytomegalovirus (hCMV) and Epstein–Barr virus in cultured immune-cell models [34,35,36]. However, gene-editing strategies have not yet been studied for antiviral targeting of VZV replication or its latent state, as far as we are aware.

In this study, the potential of gene editing to target VZV genomes as a means to prevent lytic, latent, and reactivated infections was investigated. The *Staphylococcus aureus* CRISPR/Cas9 (saCas9) system was exploited because it has a high specificity and small size, permitting efficient packaging into AAV together with guide RNA [37,38]. VZV essential and duplicated genes present in the internal and terminal genome repeat regions were targeted, allowing for the cleaving of genomes at more than one position using a single vector. The VZV genome contains three duplicated genes in the repeated sequences bounding the short unique region, ORFs 62/71, 63/70 and 64/69 [39,40]. Of these, ORFs 62/71 and 63/70 encode regulatory proteins [41,42] that have been shown to be essential [43,44]. Furthermore, ORF62 encodes IE62, the VZV ortholog of the well-characterized HSV-1 ICP4 transcriptional transactivator that is required for the expression of all HSV-1 early and late genes. The HSV-1 ICP4 activates transcription by recruiting the host-cell transcriptional machinery to the genome [45]. VZV IE62 enhances the infectivity of transfected VZV DNA [41,46] and has a sufficient functional-conserved structure to HSV ICP4 so that it can partly replace ICP4 in the HSV genome leading to the production of an infectious virus [47,48]. We selected AAV vectors for delivery because AAVs have been used both in animal models as well as clinical trials for neuronal delivery, with promising therapeutic delivery potential [49,50,51,52,53]. Here, we demonstrate that AAV2-packaged saCas9 targeting VZV ORFs 62/71 can greatly reduce VZV lytic replication in epithelial cells and lytic-infected human neurons, severely curtail VZV growth and damage the virus following reactivation from latency in neuron cultures.

## 2. Materials and Methods

### 2.1. Cells and Viruses

All cell lines except the NIH-registered human embryonic stem cell line Wa09 (H9) were purchased from ATCC. Cell-culture reagents were obtained from Thermo Fisher Scientific (Waltham, PA, USA) unless otherwise noted. The Wa09 (H9) was obtained from WiCell (Madison, WI, USA) and differentiated into neurons as detailed previously [22]. Retinal-pigmented epithelial (ARPE-19) and HEK 293 cells were grown in Dulbecco’s Minimal Essential Media (DMEM, #10569-010) supplemented with 10% fetal bovine serum (FBS; #S11150, R&D Systems, Minneapolis, MN, USA), 100 units/mL penicillin + 100 mg/mL streptomycin + 0.25 mg/mL amphotericin B as an antibiotics/antimycotic solution (#ABL02, Caisson, Smithfield, UT, USA). Human melanoma (MeWo) cells were maintained in Minimum Essential Media (MEM) supplemented with 10% FBS and antibiotics/antimycotic.

All infection studies used virus or viral recombinants based on the Parent of Oka (pOka) strain, a wildtype clinical isolate that was the parent of the live attenuated varicella and zoster vaccines. Cell-associated VZV were prepared as previously described [54], as infected ARPE-19 cells that were frozen after mitotic inhibition with growth media containing 0.01 mM mitomycin C (#A4452, ApexBio, Houston, TX, USA) for 3–4 h. at 37 °C. Cell-associated VZV was slow-frozen at −80 °C overnight in media containing 10% DMSO before long-term liquid-nitrogen storage. Frozen aliquots were titrated in triplicate for subsequent infections. Cell-free VZV was prepared using a previously published protocol [55], stored aliquoted in liquid nitrogen, and titrated after freezing for use in subsequent infections.

### 2.2. CRISPR/Cas9 Plasmid and gRNA Design

Three optimal gRNA target sequences with PAM motifs and guide length that were predicted to target saCas9 to each of ORF62 and ORF63 were selected using an online protocol from the Zhang lab [37] and Software (Benchling Inc., San Francisco, CA, USA). Oligonucleotides (Table 1) were synthesized by Integrated DNA Technologies (Integrated DNA Technologies INC., Coralville, IA, USA). Oligonucleotide annealing and cloning into the backbone vector pX601 (pX601-AAV-CMV::NLS-SaCas9-NLS-3xHA-bGHpA;U6::BsaI-sgRNA was a gift from Feng Zhang; Addgene plasmid # 61591; http://n2t.net/addgene:61591; RRID:Addgene_61591 accessed on 10 January 2022) were performed as published online (https://media.addgene.org/cms/filer_public/6d/d8/6dd83407-3b07-47db-8adb-4fada30bde8a/zhang-lab-general-cloning-protocol-target-sequencing_1.pdf accessed on 10 January 2022). Briefly, the vector was digested with BsaI and a dsDNA formed by the hybridization of the two oligonucleotides was ligated into the pX601 plasmid, where pX601 expresses saCas9 and the inserted gRNA from the same vector. A vector expressing *Streptococcus Pyogenes* CRISPR/Cas9 targeting the intergenic region between UL3 and UL4 was developed using the pX330 vector (pX330-U6-Chimeric_BB-CBh-hSpCas9 was a gift from Feng Zhang (Addgene plasmid # 42230; http://n2t.net/addgene:42230; RRID:Addgene_42230 accessed on 10 January 2022), as previously described [56,57]. Primers used for making gRNA are listed in Table 1.

### 2.3. Preparation of AAV

AAV reporter vectors of different serotypes expressing the green fluorescent protein under the chicken β-actin promoter CAG were acquired from Virovek (Virovek, Hayward, CA, USA). High titer (10^13^ viral genome copies (GC) per mL) ORF62-targeting AAV2 and a pX601 vector-only control lacking gRNA, were prepared by Penn Vector Core’s Gene Therapy Program (https://gtp.med.upenn.edu/, accessed on 10 January 2022). Lower titer (approximately 10^10^ GC/mL) control or ORF62-targeting AAV2 for use in preliminary experiments were made by transfecting HEK293 cells with the control pX601 (with no gRNA) or ORF62-targeting plasmid (pX601+62-1 gRNA), along with plasmids expressing AAV2 replication gene (pRC2-mi342 vector) and a helper plasmid that expresses adenoviral helper proteins (pHelper; Takara, Kusatsu, Shiga, Japan) using the Xfect reagent (#631318, Takara). 6 h after transfection, media was changed to fresh DMEM with 10% FBS and cells were further incubated at 37 °C 5% CO_2_ for 72 h. Cells were then harvested into an AAV lysis buffer (50 mM Tris, 150 mM NaCl, 0.05% Tween-20, pH 8.5) and subjected to three freeze–thaw cycles. AAV were then purified using a published protocol in which a discontinuous 15%–25%–40%–60% iodixanol gradient was used. Virus at the interface between 40% and 60% iodixanol was harvested after centrifugation at 200,000× *g* for 2 h. at 18 °C [58,59]. Samples were then concentrated into 0.001% Pluronic F68 with 200 mM NaCl in D-PBS using Amicon Ultra-15 centrifugal filters (#C7715, Millipore Sigma, Burlington, MA, USA) and spun at 3700× *g* for 20 min at 4 °C. AAV stocks were then aliquoted and stored at −80 °C until use. An aliquot of each virus produced was titered by quantifying AAV genomes using qPCR as detailed below.

### 2.4. Quantification of Viral Genomes

The pX601-based AAV vectors were quantified by SYBR qPCR as described previously [60], using PowerUp SYBR Green Master Mix (#25741, Thermo Fisher Scientific) and primers that amplified a 93 bp fragment of the saCas9 gene in all pX601 vectors (Table 2, “SaCas9”). Linearized plasmid DNA served as the standard, and all measurements were performed in triplicate. PCR conditions were 50 °C for 2 min; 95 °C hot start for 5 min; 45 cycles of: 95 °C for 15 s, 8 °C for 10 s and 72 °C for 45 s, followed by a dissociation curve analysis (95 °C for 15 s, 60 °C for 60 s, 95 °C for 15 s) to exclude the nonspecific amplification and formation of primer-dimers.

For quantification of VZV genomes, the PowerUp SYBR Green Master Mix was again used with primers that amplified a 60 bp fragment of the VZV ORF49 gene (Table 2, “ORF49 (VZV genome)”). DNA isolated from cell-free VZV-pOka of known genome copy number content was used to generate a standard curve for genome number. The qPCR conditions were 50 °C for 2 min, 95 °C hot start for 5 min; 45 cycles of: 95 °C for 15 s, 58 °C for 10 s and 72 °C for 45 s. Dissociation curve analyses from 60 to 95 °C were performed as described above.

### 2.5. Generation of Recombinant VZV

VZV were generated using a modified version of an established bacterial artificial chromosome (BAC) system (pOka DX), based on the VZV parental Oka strain (pOka) containing a self-excisable mini-F replicon [61,62]. The parent BAC (pOka DXRR 57Luc-ZeoR) was partly detailed elsewhere [63], and is corrected for two nonsynonymous coding mutations in ORFs 40 and 50 that differed from the sequence of the parental pOka strain. It also contained a firefly luciferase reporter fused in-frame to the ORF57 gene using T2A ribosome skipping motif, and is expressed from the ORF57 late promoter (PRK and MBY, manuscript in preparation). The BAC was manipulated using two-step, markerless λ-Red-mediated recombination as previously described [61,62] in the E. coli strain GS1783 (a gift from Dr. Gregory Smith, Northwestern University, Chicago, IL, USA). The GS1783 contains a cassette of L-arabinose-inducible expression of the ISceI homing restriction enzyme and the 42 °C heat-inducible expression of the λ-Red recombination genes. All PCR amplifications for cloning and recombineering were performed using high fidelity PrimeSTAR GXL polymerase (#R050B, Takara). Final manipulated BACs were evaluated by restriction fragment length polymorphism (RFLP) analyses and BACs made into viruses were sequenced across the relevant sites of insertion and/or mutagenesis.

A VZV dual reporter BAC and virus were developed in which the fluorescent mCherry reporter gene was fused to the N-terminus and in-frame with ORF23, which encodes a minor capsid protein. This was derived by PCR-amplification from the plasmid pmCherry-kan, which contained a reversable ISceI site containing a kanamycin resistance cassette (kan^r^) derived from the plasmid pEPS-kan2 (a gift from Dr Gregory Smith, Northwestern University, Chicago, IL, USA). Resolving the internal kan^r^ cassette by inducing a further recombination concurrent with the induction of ISceI expression resulted in BACs with restored mCherry-ORF23. The VZV derived from this BAC was termed VZV DR (dual reporter) and expressed mCherry from the ORF23 promoter and luciferase from the ORF57 promoter. Then, the ORF71 coding sequence was replaced with a PCR-amplified ampicillin resistance cassette (amp^r^), selecting for gain of ampicillin resistance in addition to resistance to chloramphenicol (chm^R^ is directed by the replicon) and zeomycin resistance (directed by a cassette inserted downstream of the luciferase at ORF57). VZV derived from this new BAC (VZV DR-Δ71) contained two quantifiable reporters and one copy of the ORF62 gene. Evaluation of virus at passage 6 by Southern blotting confirmed that the virus contained a restored ORF71 derived from ORF62 gene (VZV-DR-Δ71: data not shown). The VZV BAC DR-Δ71 was then further subjected to a recombinatorial mutation of the ORF62 gRNA-targeted sequence region to derive VZV DR-62gmut mutant BACs and viruses. Two primers were used to amplify kan^r^ from pEPS-kan2 (Table 3, “gRNA region primers”). These changed eleven base pairs of the DNA sequence of ORF62 targeted by the 62-1 gRNA, without altering the encoded ORF62 amino acids (Table 3 “Wobble base pair changes”). The PCR amplified DNA was recombined into ORF62 in the BAC VZVΔ71, then selecting BACs for gain of kanamycin, chloramphenicol, and zeomycin resistance. RFLP was used to identify BACs and the kan^r^ was reversed out of the BAC by a second recombination and the induction of ISceI expression, as detailed elsewhere [61,62]. Sequencing across the region in the BAC and of the resulting virus after passage 6 confirmed the presence of the desired engineered mutations.

### 2.6. Virus Derivation and Growth Curves

The virus was generated from the BACs that were just detailed after their transfection into MeWo cells, using Lipofectamine 3000 (#L3000015, Thermo Fisher Scientific) with 2.5 μg of purified BAC DNA and 100 ng each of plasmids expressing ORF61 and ORF62 DNA under the constitutive hCMV immediate-early promoter, as detailed previously [64,65]. Upon the appearance of fluorescent plaques, infected cultures were amplified by trypsinization and replated with uninfected ARPE-19 cells until they exhibited >80% cytopathic effect. Viruses were further amplified for a minimum of six passages to self-excise the BAC replicon elements and restore ORF71. Virus stocks were generated from ARPE-19-infected cultures showing >80% cytopathic effect, after treating cultures with 0.01 mM mitomycin C for 3–4 h. at 37 °C prior to harvesting for cryopreservation, as detailed previously [65].

Virus growth curves were determined as detailed previously [64,65], initiating infections in confluent 6-wells with approximately 400 pfu of virus (per 0.5 × 10^6^ cells in a single well) from cryopreserved, pre-titered, mitomycin C-treated aliquots of infected ARPE-19 cells. All assays were performed in triplicate. At the desired times after infection, infectious virus-associated cells were quantified by trypsinizing the monolayers and then seeding serial dilutions onto ARPE-19 monolayers grown in 6 wells seeded 24 h. prior and at 80% confluency. At 4–5 dpi, the formed plaques were counted under a fluorescent microscope, and averaged from the triplicate values. Titers were normalized to the exact titer of virus at day 0 determined from the inoculate and then normalized to parental virus VZV DR.

### 2.7. Neuron Cultures

Neuron cultures were prepared using a previously published protocol [22] based on the differentiation of H9 human embryonic stem cells (hESC) cultured on feeder cells for 1–2 weeks. H9 cells were then further amplified on a feeder-free platform. This used StemFlex^TM^ medium supplemented with StemFlex^TM^ supplement (Thermo Fisher Scientific) and antibiotic/antimycotic. Cells were grown on 6-well dishes pre-coated with GelTrex Basement Membrane Mix (#A1413302, Thermo Fisher Scientific). Colonies were passaged either by manual dissection or using ReLeSR™ Enzyme-free cell selection and passaging reagent (Stemcell Tech., Vancouver, BC, Canada), per the manufacturer’s instructions. Following either method, ROCK inhibitor Y-27632 (#S1049, Selleckchem, Houston, TX, USA) was added to the culture media for 24 h. to increase cell survival and deplete dividing cells. Neural precursor cells (NPCs) were generated from hESC by co-culture with the PA6 mouse stromal fibroblast cell line (RIKKEN BioResource Center, Tsukuba, Ibaraki Province, Japan) as detailed previously [66,67]. For terminal differentiation, neurospheres were added to culture dishes coated sequentially with Poly-D Lysine (PDL) and GelTrex and cultured for 14 days in a Neuron Medium (Neurogenic Medium supplemented with CultureOne (#A3320201, Thermo Fisher Scientific).

For the fluorescent monitoring of VZV infections of neurons, neurospheres were seeded on coated 12-well glass bottom plates (P12-1.5P, Cellvis, Mountainview, CA, USA) and differentiated in neuron differentiation media for at least 14 days. Infections were initiated with PBS-washed, mitomycin-C treated VZV-infected ARPE-19 cells. Medium was changed the next day and every 2–3 days after. Latent infections were initiated with cell-free virus stocks of a previously detailed recombinant VZV (VZV66GFP) that expressed ORF66 as a GFP-ORF66 fusion [22,68] Latently infected neurons were then maintained in media containing 50 µM acyclovir (ACV, #A1915, Tokyo Chemical Industry, Tokyo, Japan) to block sporadic lytic initiation events, as previously described [23]. Latent infections were monitored for at least one week after infection in the absence of ACV by fluorescent microscopy screening to remove from consideration any cultures showing breakthrough GFP expression as a marker for productive infection. For reactivation, the media was then replaced with the same media lacking ACV and NGF, but including 50 μg/mL anti-NGF (Biolegend, 617904) and 50 ng/mL 12-O-tetradecanoylphorbol-13-acetate (TPA; #1201 Tocris Biochemicals, Bristol, UK). Reactivation was monitored for 1–2 weeks by microscopic screening for GFP expression. To quantify the reactivated virus, treated neurons were dissociated by manual dislodging and trituration, then by replating dilutions of the neurons onto confluent ARPE-19 monolayers. Foci of infection were counted 4 days later, using a fluorescent microscope.

### 2.8. Fluorescent Microscopy

Live neuronal cultures were monitored using a Nikon TI fluorescent microscope with a 10× air objective (N.A. 0.30). Imaging of fixed neuron cultures followed previous detailed procedures [22,23]. Neurons were immunocytochemically identified by chicken anti-beta tubulin III (Novus Biologicals, Moon Township, PA, USA) in 10% heat inactivated goat serum (HIGS) in PBS. Bound antibodies were detected using secondary goat anti chicken antibodies linked to AlexaFlor-594 (#A-11042, Thermo Fisher Scientific,). Cultures were then mounted in mounting media containing 4′,6-diamidino-2-phenylindole (DAPI) to stain nuclei. Images for all samples in each experiment were captured under identical acquisition settings and processed using Metamorph software (Version 7.7, Molecular Devices, San Jose, CA, USA). VZV fluorescent foci in infected epithelial cells were imaged at 4 dpi after growth at 34 °C after fixing in 4% paraformaldehyde and washed with 1× PBS. Multiple images containing entire individual foci that did not touch any borders or other plaques were acquired under identical acquisition settings with CellSens software (Olympus, Tokyo, Japan) on an Olympus IX83 microscope with a 10× (N.A.030) air objective. Data was exported and analyzed for size using Metamorph.

### 2.9. Flow Cytometry and Statistical Analyses

Flow cytometry was used to quantify GFP-fluorescent positive HEK293 cells in the initial evaluation of the efficiency of gRNAs to target genes and block protein expression. All flow cytometry samples were collected and analyzed using a BD FACSAria cytometer (Becton, Dickenson, and Co., Franklin Lakes, NJ, USA) and FlowJo software (FlowJo, Ashland, OR, USA). Statistical analyses were performed using GraphPad Prism software (GraphPad, La Jolla, CA, USA). Where indicated, error bars represent standard deviation (SD) or standard error of the mean (SEM) as specified in the figure legends.

## 3. Results

### 3.1. Characterization of VZV-Specific gRNAs for In Vitro Specificity

The goal of the studies was to assess the potential of using gene editing as an anti-viral strategy targeting VZV, not only in lytic-infected cells that are permissive for VZV, but also in reactivating neuron cultures. We recently detailed a hESC-derived neuron culture system that can host a VZV model latent state that can subsequently be reactivated [23]. Gene editing has potential to target the latent genome before reactivation. Gene editing strategies targeting HSV-1 have suggested that two sites of double stranded breaks (DSBs) are more efficient in reducing the HSV genome load and progeny virus production compared to single genome DSB sites, which can repair. The latter can still be disruptive as a consequence of errors in DNA repair that result in indels in a single-copy critical gene [33,69]. Our gRNA-based saCas9 antiviral strategy targeted duplicated essential VZV genes. Both ORF63/70 and ORF62/71 lie in the large reiterated genomic regions bounding the short unique region. This region also contains the ORF64/69 gene pair, but this pair has been shown to be dispensable for in vitro replication in culture, and in human T cells and human fetal skin that is harbored in SCID-hu mice [43]. VZV ORF 62/71 protein functions as the major transcriptional activator (transactivator) in VZV [41], while ORF63 protein has regulatory [70] and anti-apoptotic [71] activities, and ORF63 transcription is associated with latency [72]. One report has suggested that ORF63 is not essential for VZV culture growth [73], while others report ORF63 as absolutely required for VZV replication [43].

**Figure 1:**
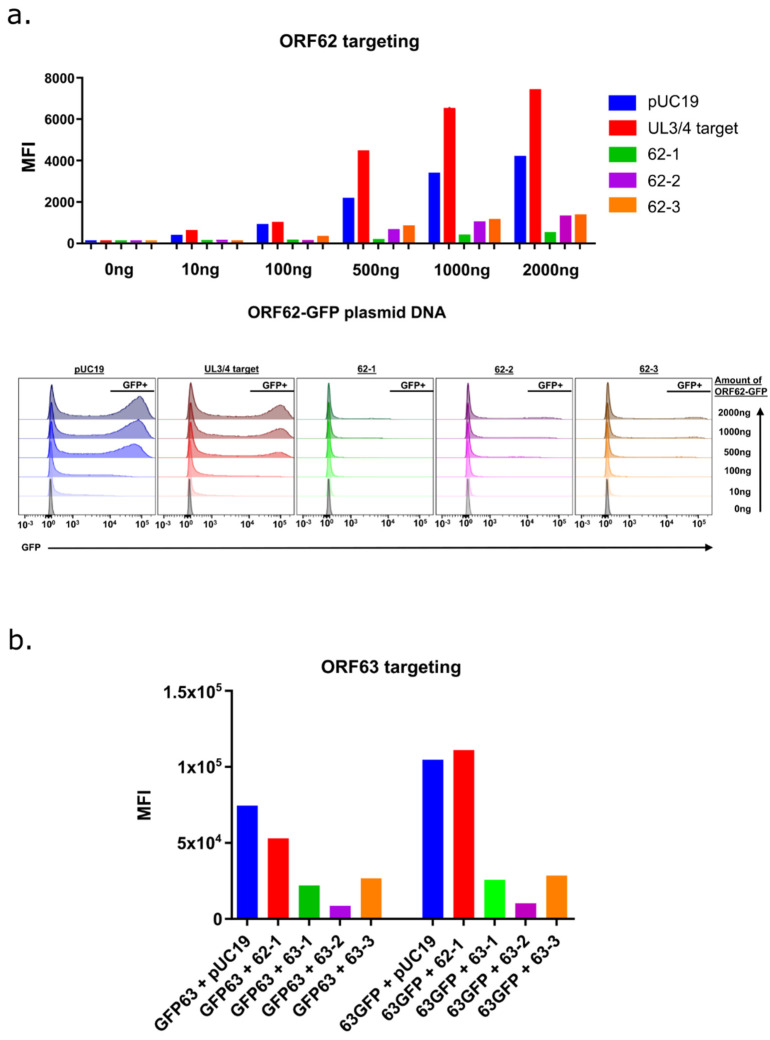
A preliminary study to evaluate ORF62- and ORF63-targeting gRNAs in pX601 to prevent gene expression. (**a**) HEK 293 cells in 6-well plates were transfected with a range (0–2000 ng) of CMV-ORF62-GFP plasmid, along with 2 μg of plasmid pUC19; a pX330-based plasmid with a gRNA template derived from HSV UL3/4; or pX601 AAV plasmids containing the templates for ORF62 gRNAs 1–3, constructed as detailed in the Methods. To maintain the same level of DNA in each transfection, pUC19 was added. At 36 h post transfection, cells were harvested by trypsinization and analyzed by flow cytometry to determine the mean fluorescence intensity (MFI) for each treatment. (**b**) To determine the optimal gRNAs for targeting ORF63 in pX601, HEK293 cells in 6-wells were co-transfected with 500 ng of CMV-promoter-driven ORF63-GFP plasmid expressing a C-terminal tagged GFP (63GFP) or a similar plasmid-driving expression of N-terminal tagged GFP (GFP63); each with either 1500 ng of pUC19 DNA, the px601 containing the 62-1gR template, or one of three selected pX601 plasmids containing the ORF63-gR templates as detailed in Table 1. After 72 h, the cells were subject to flow cytometry and the MFI was determined using FlowJo software. The data represent the MFI of single transfections performed in parallel and were not statistically evaluated.

Three gRNA candidates for each gene were selected based on an optimal on/off-target specificity, the presence of the PAM motif, and length. The appropriate hybridized oligonucleotides were then cloned into the AAV-based pX601 backbone plasmid (Table 1) co-expressing saCas9. We then conducted a preliminary study to demonstrate that each gRNA construct was able to target the respective gene. Each pX601-based plasmid or control (pUC19 and pX330-UL 3/4) plasmid was co-transfected with corresponding CMV IE promoter-driven ORF-GFP fusion constructs into HEK293 cells. We reasoned that cleavage by the targeted saCas9 should prevent translation of the protein and reduce expression of the GFP reporter, measured by flow cytometry. Comparison of the three ORF62-specific gRNA constructs revealed that each greatly reduced the expression of GFP from the CMV-62GFP reporter as compared to control plasmid co-transfected cells (Figure 1a). A similar but slightly different protocol was then used to evaluate ORF63-targeting gRNAs, in which a single set amount of CMV-ORF63-GFP fusion (500 ng) was co-transfected with control plasmids pUC19 or pX601 containing the 62-1gRNA or the 3 different 63gRNA templates. All of the 63gRNA showed a reduced level of GFP expression from both ORF63 N-terminal (GFP ORF63)- or C-terminal (ORF63 GFP)-tagged expression plasmids, when compared to the non-targeting CRISPR/Cas9 constructs. Taken together, the data suggested that all gRNAs could efficiently target the respective gene. We then selected the “62-1” ORF62-targeting construct (62-1gR-saCas9) for further development.

### 3.2. AAV Serotypes for Delivery to VZV Permissive Epithelial Cells and Human Stem Cell-Derived Neurons

**Figure 2:**
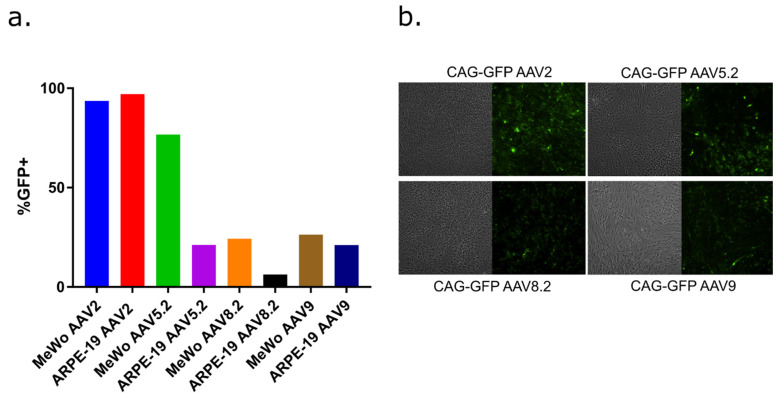
A preliminary study of AAV serotypes able to deliver to epithelial cells and neurons. (**a**) The fraction of two VZV permissive epithelial cell lines (MeWo and ARPE-19) that expressed GFP, as determined by flow cytometry, after infection with 5 µL of 10^12^ GC/mL AAV per well of a 6 well plate with AAV-GFP serotypes 2, 5.2, 8.2 or 9. AAV-mediated expression was measured by flow cytometry at 3 days post transduction and is represented as a fraction of the total cell population, using gates selected by analyses of untransduced cells. Of the four serotypes evaluated on MeWo and ARPE-19 cells, AAV-2 was the most efficient at transducing both cell types to express GFP. The study represents single transductions and was not statistically evaluated. (**b**) To qualitatively establish the transduction of hESC-derived neurons, neurons cultured in 12-well dishes were transduced with 1.25 × 10^11^ GC per 12-well of each GFP-expressing AAV serotype. The AAV-mediated expression of GFP was imaged at multiple (>5) non-overlapping random positions at 6 days. Representative images are shown that reflect GFP expression in neuron cultures. Further quantification could not be determined due to the heterogeneous and three-dimensional nature of the neuron cultures.

AAV capsid serotypes confer different tissue tropisms [74,75,76,77]. We considered it important to identify an AAV serotype that transduces epithelial cell lines that support VZV replication, in addition to being able to transduce hESC-derived neurons that can harbor model VZV reactivatable latent states. A preliminary study was conducted with four commercially acquired AAV neurotropic serotypes containing CAG-GFP reporters (serotypes 2, 5.2, 8.2, and 9), which have been previously exploited for murine-neuron delivery [78]. AAV 5.2 and 8.2 recombinant serotypes contain modifications of the corresponding WT serotypes that are potentially better able to escape cellular vesicles once endocytosed, avoiding lysosomal breakdown (Virovek, Hayward, CA, USA). AAV transduction with each serotype was performed at equivalent genome copy levels per cell and the transduction of MeWo and ARPE 19 cells was assessed by determining the fraction of cells showing GFP expression, using flow cytometry after 3 days incubation (Figure 2a). AAV2 appeared to be efficient in transducing both MeWo and ARPE19 cells, as GFP expression was detected in nearly 100% of the cells for both lines. Other serotypes did not result in GFP expression in the majority of cells of both lines. The ability of each serotype to transduce and express GFP in hESC-derived neurons was also confirmed. Fluorescent microscopy images (Figure 2b, enlarged in Supplemental Figure S1) indicate that all four serotypes resulted in GFP expression in the hESC-derived neuron culture platform. For AAV2, the GFP intensity appeared weaker than that seen after neuron transduction with AAV5.2, but AAV5.2 appeared to transduce a lower proportion of neurons compared to AAV2.

These observations are consistent with previous studies reporting the ability of AAV2 and AAV5 to transduce ARPE-19 cells [79] and murine neurons [80]. The three-dimensional nature of hESC-derived neuron cultures [22] did not permit more accurate quantitation of neuronal transduction, but given the ability of AAV2 to efficiently transduce VZV permissive epithelial cells close to 100%, we selected AAV2 for packaging of the pX601-based vectors containing saCas9, with or without the 62-1 guide RNA.

### 3.3. AAV-62-1gR-saCas9 Reduces VZV Lytic Replication in Epithelial Cells

High-titer preparations of AAV2-packaged saCas9 with (AAV-62-1gR-saCas9) or without (AAV-saCas9) the 62-1 gRNA sequence template were obtained at more than 10^12^ GC/mL and subsequently evaluated for their ability to prophylactically reduce lytic replication in VZV-infected ARPE-19 cells. ARPE-19 were mock treated or transduced with each AAV at 10^4^ GC/cell and 4 days later, cultures were infected with a low dose (500 pfu per well of a 6-well plate) of mitomycin C-treated, cell-associated VZV DR or VZV DR-Δ71 (see Methods in Section 2). Southern blot analyses of DNA from VZV DR-Δ71 after 6 passages in culture established that ORF71 in the virus was restored by the reduplication of ORF62 (data not shown), as expected from similar previous mutagenic studies of ORF62 in the BAC from our group [64]. Parallel infected cultures were harvested multiple times after VZV infection and assessed for the expression of the kinetically late ORF57 promoter-expressed luciferase activity.

**Figure 3:**
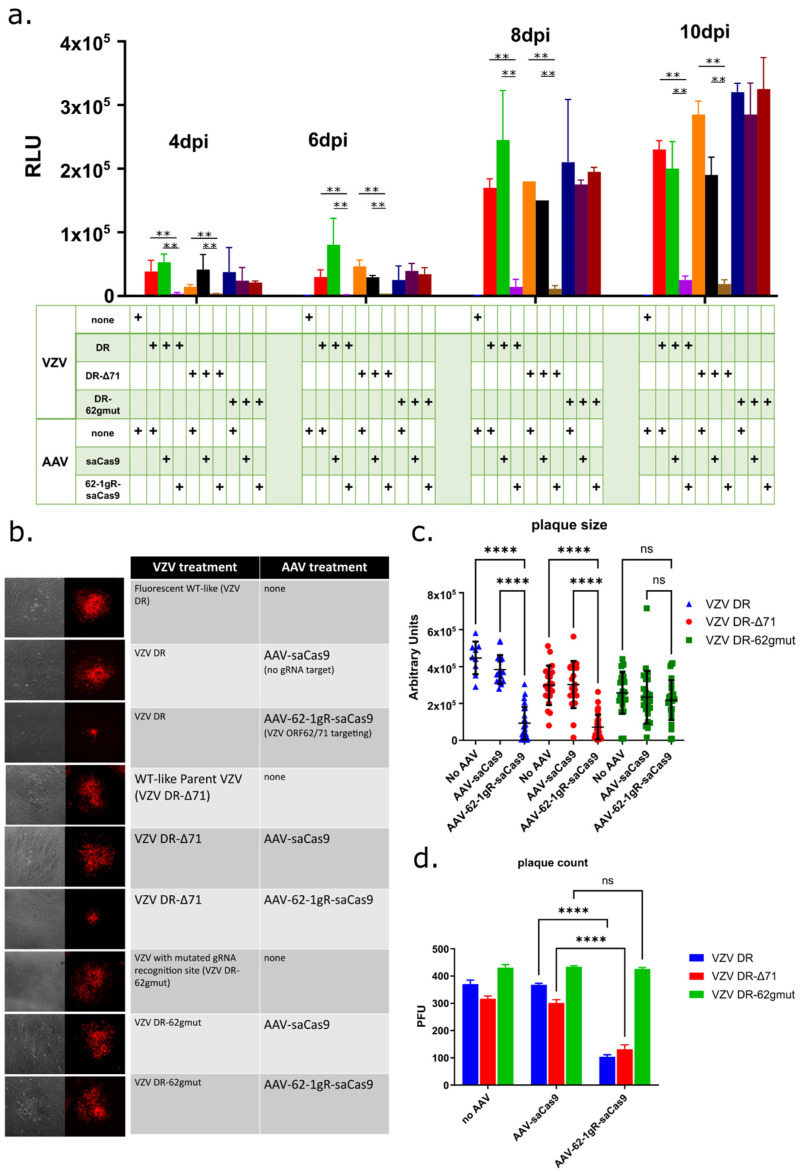
AAV-62-1gR-saCas9 reduces VZV productive infection in infected epithelial cells. (**a**) The 12-well plates of near confluent ARPE-19 cells were pre-treated with 10^4^ GC/cell of the different AAV and at 4 days post transduction, cells were infected with 500 PFU/well of cell-associated VZV. VZV replication was measured using luciferase-reporter expression from the ORF57 gene at 4, 6, 8, and 10 days after VZV infection. Cell extracts were diluted 1000-fold prior to the assay. Significant differences between treatments, defined as *p* < 0.01 using a 2-way ANOVA with Geisser-Greenhouse’s correction, are indicated by two asterisks (**). The box below the graph illustrates the components added to each condition and each condition was performed in triplicate. RLU = Relative Light Units (**b**) Representative images of infectious foci by phase-contrast imaging (first column, in gray) and by live cell fluorescence for the ORF23 promoter-driven mCherry (second column, in red) at 4 days after VZV infection in ARPE-19 cells. The boxes to the right illustrate the virus (third column) and the AAV (fourth column). (**c**) Quantitation of fluorescent focus size determined from at least 9 and less than 31 isolated, nonoverlapping foci measured for each sample condition (**** = *p* < 0.0001, 1-way ANOVA with Tukey’s multiple comparisons test). The average size is indicated by the horizontal bar. (**d**) Average visible focus counts per 6 well that were observable under 10× objective. The number of foci/well (bars represent counts from triplicate wells) were clearly reduced in AAV-62-1gR-saCas9-pretreated samples infected with WT-like VZV, compared to cells infected with vectors expressing SaCas9 without guide RNA (“AAV-saCas9”) (**** = *p* < 0.0001, 2-way ANOVA with Tukey’s multiple comparisons test). All data shown is from one of two experiments with similar results. Error bars represent STD. ns = not significant.

In ARPE-19 cells not pretreated with AAV before VZV infection, or in cells pretreated with AAV-saCas9 (no guide RNA), VZV luciferase activity reported an increase in growth of both VZV DR and VZV DR-Δ71 over time. Strikingly and in contrast, cells pretreated with 10^4^ GC/cell of ORF62-targeting AAV showed a dramatic reduction in the VZV reporter activity over time as compared to untreated and AAV-saCas9 controls for both viruses. This indicated that AAV-62-1gR-saCas9 was highly effective at reducing progeny virus production (Figure 3a). The VZV derived from a BAC in which ORF71 was deleted gave very similar results to VZV DR, as expected. An assessment of viral growth visualized by mCherry expression in infected foci (Figure 3b) supported the luciferase studies, in that both focus size (Figure 3c) and the number of focus numbers at 4 dpi (Figure 3d) were vastly reduced in cells pretreated with AAV-62-1gR-saCas9, compared to controls, for both VZV DR and VZV DR-Δ71. These data indicate that the AAV-62-1gR-saCas9 blocks lytic replication of VZV in infected epithelial cells. Furthermore, the reduction in foci numbers developing in AAV-62-1gR-saCas9 pretreated cells strongly suggests that some infected cells failed completely to initiate the productive spreading foci of infection.

**Figure 4:**
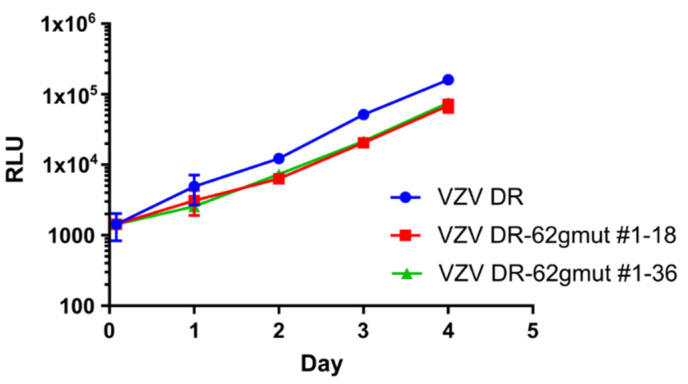
VZV DR-62gmut grows similarly to WT VZV. Naïve ARPE19 cells were infected with equivalent amounts of VZV-infected ARPE-19 mitomycin C treated cells, as detailed in the methods. Luciferase activity was measured daily. Graphs show the growth of two independently isolated clones (“#1–18” and “#1–36”) of VZV DR-62gmut and VZV DR WT-like reporter virus. Each point represents the average of four replicates and the data shown is representative of two independent experiments. Error bars represent STD.

The AAV-62-1gR-saCas9 was designed to specifically target VZV at the ORF62/71 gene, but a well-known issue with gene editing technologies is the possibility of off-target effects. It is conceivable that a cellular factor required for VZV replication was damaged by off-target activities that would result in similar observations to those reported above. Therefore, as an additional control, two VZV recombinants in the background of VZV DR-Δ71 were derived, in which the guide RNA target sequence was mutated at 11 bases in the codons of ORF62 (VZV DR-62gmut clones #18 and #36) that maintained the same encoded open reading frame residues for ORF62. The expectation was that the substitutions of 11 bases in the Cas9 recognition region of ORF62 would render the 62-1 gRNA no longer able to recognize and cleave at this location (Table 3). If the effect was acting though off-target activities, it would still impair the viral replication of such viruses. Growth curve analyses of the two VZV mutants after low-MOI infection of ARPE-19 monolayers indicated that the rate of luciferase expression over time was only slightly less than that of VZV DR, suggesting the silent mutation of codons might have a very minor influence on growth (Figure 4). However, testing the growth of one of these viruses (VZV DR-62gmut # 1–18) in the same conditions as those used in the experiments shown in Figure 3 revealed that in AAV-62-1gR-saCas9-pretreated cells, the mutant VZV grew at rates similar to that in cells that were not AAV-pretreated or pretreated with the AAV-saCas9 control. The mutant virus did not show the dramatic reduction in luciferase activity, plaque size and plaque number as seen for the parental VZV DR and VZV DR-Δ71 viruses. These experiments strongly suggest that the reduction of VZV replication by pretreatment with AAV-62-1gR-saCas9 was a consequence of the specific targeting of VZV ORF62/71 DNA.

### 3.4. AAV-62-1gR-saCas9 Reduces VZV Lytic Replication in hESC-Derived Human Neuron Cultures

**Figure 5:**
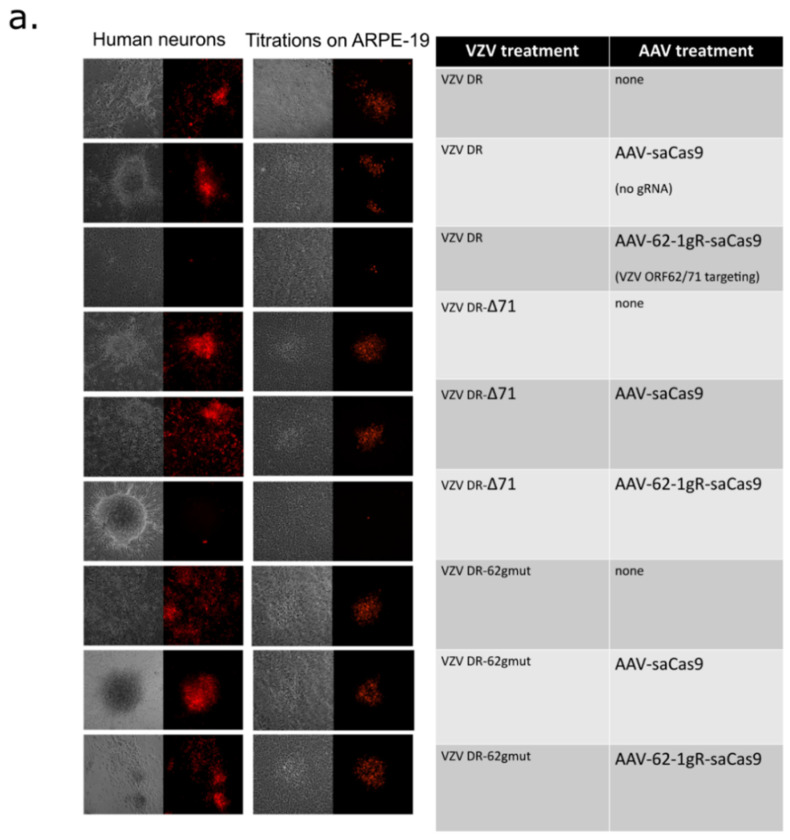

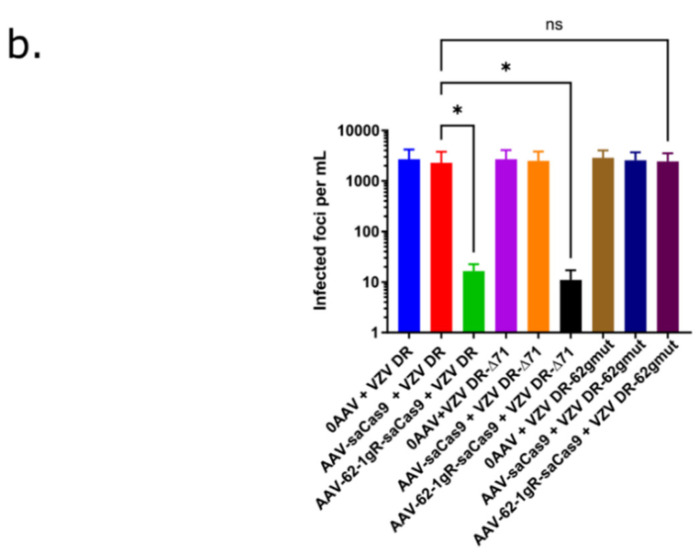
Pretreatment with AAV-62-1gR-saCas9 knockdown of VZV lytic replication in exogenously infected human neuron cultures. (**a**) The 12-well plates of hESC neuron cultures were transduced with 104 GC/cell of AAV-62-1gR-saCas9 constructs/well. Four days later, neurons were infected with 500 PFU of mitotically inhibited fluorescent (red)-cell associated VZV-infected cells to initiate lytic infections. Infections were subsequently monitored by fluorescence microscopy and representative images of mCherry reporter expression of infected cells and the same fields observed by phase contrast microscopy were acquired 10 days after VZV infection. (**b**) After imaging, duplicate infected neuron cultures were scraped, triturated, and then seeded onto confluent ARPE-19 cells to quantify the number of VZV-infected neurons by the number of infectious foci they generated. Foci were counted after 5 days. Representative images of foci formed on ARPE-19 at day 5 from virus obtained from the treated neuron cultures are shown in column 2 of Figure 5a. Data represents quadruplicate measurements and significant differences between treatments depicted by asterisks (* = *p* < 0.01, ordinary one-way ANOVA with multiple comparisons; ns = not significant). Similar results were obtained in two independent experiments. Error bars depict the STD.

The ability of AAV-62-1gR-saCas9 treatment to block lytic replication in hESC-derived neurons was then evaluated. We have previously shown that hESC-derived neuron cultures are able to support the spreading of productive VZV infections [22,23,81]. Neurons that were differentiated from neurospheres for 3 weeks were transduced with approximately 10^4^ GC/cell of AAV or left untreated. Four days later, the cultures were infected with 500 PFU of mitotically inhibited VZV DR-infected ARPE-19 cells. Viral growth was monitored visually at the same position in the cultures by microscopy over a 10-day period, and representative live-cell images of the foci of fluorescence in the neuron cultures at day 10 post infection are shown in the first column of Figure 5a. In non-AAV treated, AAV-saCas9 pre-treated and AAV-62-1gR-saCas9 pretreated neurons infected by VZV DR-62gmut, foci of VZV infected neurons (indicated by red fluorescence) clearly developed over the 10-day incubation period (Figure 5a). However, neuron cultures treated with AAV-62-1gR-saCas9 that were infected with VZV DR or VZV DR-Δ71 developed only very small foci of red fluorescence that involved only a few neurons, far fewer than seen in the controls. This suggested the virus in this group was not able to spread and form foci as efficiently as infected neurons in wells receiving other treatments (Figure 5a). To obtain a more quantitative assessment, the infected neuron cultures at 10 dpi were dislodged, triturated, serial diluted and re-seeded onto monolayers of ARPE-19 cells. Infectious centers that formed from all non-AAV-treated or control AAV-saCas9-pretreated neurons after 5 days were approximately the same size, as were the infectious centers formed from AAV-62-1gR-saCas9 pretreated cultures infected with VZV DR-62gmut virus. However, plaques formed on ARPE-19 cells co-cultured with neurons that were infected with VZV DR or VZV DR-Δ71 after pretreatment with AAV-62-1gR-saCas9 were not only dramatically reduced in number, but at the highest concentration of neurons seeded onto ARPE-19 monolayers, only tiny foci of infection involving a few fluorescent cells were seen. No wildtype-sized plaques developed after neuron seeding on ARPE-19 under these conditions. Even taking such small foci of infection as positive, the number of infectious centers for VZV DR and VZV DR-Δ71 on neurons pretreated with AAV-62-1gR-saCas9 were more than a hundred-fold fewer compared to the non AAV-treated, mock AAV-treated, or VZV DR-62gmut-infected controls (Figure 5b). The strikingly reduced size of the plaques formed by progeny virus after AAV-62-1gR-saCas9 treatment suggested that virus produced from treated neurons was unable to replicate to wild type levels. These results strongly suggest that AAV-62-1gR-saCas9 pretreatment not only efficiently reduces the production of the progeny virus in infected neurons but also, when virus is produced, it is damaged and severely impaired for further replication.

### 3.5. AAV-62-1gR-saCas9 Reduces VZV Replication Following Reactivation in Model Latently Infected Neuron Cultures

**Figure 6:**
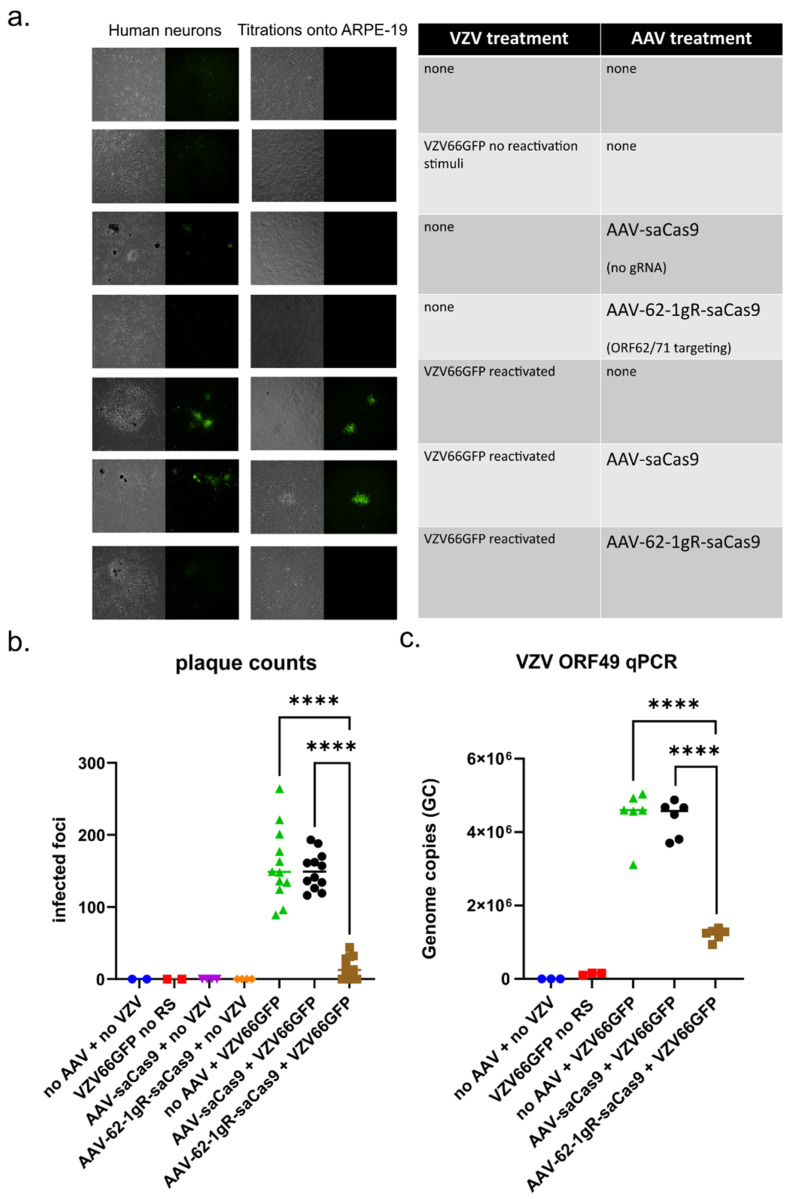
AAV-62-1gR-saCas9 treatment of latently infected neurons reduces VZV progeny and spread following reactivation induction. (**a**) hESC-derived neurons in 12-well plates were infected with 500 PFU of cell-free virus expressing GFP (VZV ORF66 GFP) in the presence of 50 µM acyclovir (ACV) to establish latent infections. After 7 days in the presence of ACV, approximately 10^4^ GC/cell of different AAV (or mock transduction) was added to the appropriate wells. ACV was then removed, and cultures incubated for a further 7 days. At 7-day post AAV transduction, GFP-negative neuron wells were left untreated or were stimulated to reactivate latent genomes by changing to neuron growth media lacking NGF and containing 50 μg/mL anti-NGF and 50 ng/mL TPA. Reactivation was then monitored by fluorescent microscopy and the representative images of the cultures were acquired at 7 days after the reactivation stimulus. Neurons were then scraped, triturated, and seeded onto ARPE-19 cells not treated with AAV, and infectious centers forming on ARPE-19 monolayers at 4 days from each of the experimental conditions were imaged (last two columns). (**b**) The number of foci forming on ARPE-19 monolayers in each 12-well plate were then counted and are shown as plaque-forming units. Each point represents the count from equal proportions of individual neuron cultures (**** = *p* < 0.0001, ordinary one-way ANOVA with multiple comparisons). (**c**) DNA was extracted from 1/3 of each neuron culture treated under the different conditions, and VZV DNA was quantified using a SYBR-based qPCR using primers against a region of VZV ORF49 (Table 2). Copy number values were determined by comparison to a standard curve determined using known concentrations of VZV genomes (with a range of 10^1^–10^7^ genome copies). Data is representative of results from two independent experiments with similar results. RS = reactivation stimuli.

A goal of VZV targeting by gene editing is to reduce the capacity of latent genomes to reactivate and induce zoster disease, not only by reducing spread at the periphery, but also in the ganglia, to reduce its intra-ganglionic spread after a reactivation event has initiated. Even without complete elimination of reactivation, a reduction of lytic replication in the ganglia could potentially limit sensory damage caused by VZV reactivation. The hESC neuron cultures used here have been shown to support a latent VZV infection (defined by the prolonged absence of any indicators of productive infection and lytic gene reporter expression), which can then be experimentally reactivated (to renew lytic reporter gene expression and produce virus that spreads to other neurons) [23]. We thus explored the potential of AAV-62-1gR-saCas9 as a treatment for preventing VZV reactivation and subsequent replication. Latent infections were established by exposing neuron cultures to cell-free virus of a previously characterized recombinant VZV that expresses GFP linked to ORF66 (VZV66GFP), and then incubated in the presence of 50 μM acyclovir (ACV) to inhibit lytic replication for 7 days. Cultures were then incubated for 7 days in the absence of ACV. None of the cultures contained GFP fluorescence indicating lytic infection. Cultures containing latently infected neurons were then transduced with approximately 10^4^ GC/cell AAV or mock transduced, and then incubated in media without ACV. At 7 days post AAV transduction, GFP positive cells were again not observed, indicating that the AAV transduction itself did not induce VZV reactivation. A set of cultures was reactivated by treating with a combination of NGF withdrawal, antibody-mediated NGF depletion and the addition of 50 ng/mL 12-O-tetradecanoylphorbol-13-acetate (TPA), and cultures were monitored daily for GFP fluorescence indicating reactivation events for 7 days. After imaging, the neurons were dislodged, triturated, and seeded onto confluent ARPE-19 cells for infectious center assay (Figure 6b).

None of the neuron cultures latently infected with VZV66GFP and not subjected to reactivation stimuli expressed GFP. When such neurons were seeded on ARPE19 cells, infectious centers did not form. In contrast, the latently infected neurons receiving the reactivation stimulus developed numerous GFP-positive foci and formed multiple infectious centers when seeded onto ARPE-19 monolayers, indicating that productive reactivation had occurred (Figure 6a and Supplemental Figure S2). Latently infected neurons treated with AAV-saCas9 without guide RNA before receiving the reactivation stimulus also developed multiple GFP positive plaques in the neuronal cultures and generated infectious foci on ARPE-19 cells. Importantly, in latently infected cultures pretreated with AAV-62-1gR-saCas9 prior to receiving a reactivation stimulus, there were no visible GFP positive centers of infection forming, even at 7–10 days post stimulus. Subsequent seeding of these neurons onto ARPE-19 cells did result in the formation of a few small GFP positive foci, but these were significantly fewer and considerably smaller in size (Figure 6a, second column; enlarged in Supplemental Figure S2). These data indicate that reactivation events were not completely prevented and virus still formed after treatment with AAV-62-1gR-saCas9, the AAV treatment greatly reduced the number of infectious progenies after reactivation induction.

To investigate whether the reduction in the number of productively infected neurons in reactivated cultures pre-treated with AAV-62-1gR-saCas9 was due to a reduction in the number reactivating genomes or a result of reduction of viral spread to additional neurons, SYBR-based qPCR quantification of VZV genomes was performed. There was an increase of more than 10-fold in genome copies at 21 days-post VZV infection in reactivated samples with or without AAV pretreatment compared to neurons not receiving a reactivation stimulus. While AAV-62-1gR-saCas9 pretreatment resulted in a significantly reduced level of genomes as compared to controls, levels of genomes measured were still higher than those seen in latently infected cultures not receiving any reactivation stimulus (Figure 6b,c). These data indicate that AAV-62-1gR-saCas9 pretreatment effectively reduced the viral burden after reactivation, but did not indicate if the latent genome load was reduced by the treatment. Thus, AAV-CRISPR/Cas9 strategy for targeting duplicated VZV genes is clearly an effective antiviral approach, greatly reducing the production of progeny by damaging genomes.

## 4. Discussion

The goal of this study was to establish proof of principle that the CRISPR targeting of VZV genomes could be used as an antiviral strategy eventually leading to therapeutic applications. The results obtained here add to a growing body of knowledge in which several human DNA viruses (and RNA viruses with DNA genome intermediates) have been targeted by gene editing [31,33,57,82,83,84]. While gene editing can be performed with designer meganucleases and transcription factor-like endonucleases (TALENS), the complexity of their design and expense of their generation has made the more simple and widely available RNA-guided CRISPR/Cas technology more attractive. CRISPR/Cas also has the advantage that multiple sites on the viral genome can be targeted by increasing the number of gRNAs, rather than needing to express two or more enzymes. CRISPR-based genome editing been used to target viruses including hepatitis B virus (HBV) [85,86], human immunodeficiency virus (HIV) [87,88], and several herpesviruses [89] such as EBV [90], CMV [91], Kaposi’s Sarcoma Virus [92], and HSV-1 [31,32,33]. In the case of EBV, antiviral gene editing has targeted both the viral genome [90] as well as key cellular components required for EBV replication [93]. To our knowledge, CRISPR/Cas-mediated antiviral targeting has not yet been applied to VZV, although it has been used as a research tool to generate recombinant VZV [94].

Here, we demonstrated efficient antiviral activity using AAV-delivered gRNA targeting a duplicated gene to reduce VZV replication, spread and virus production in both lytic-infected epithelial cells and hESC derived neurons. Importantly, we further showed that it dramatically reduced viral replication upon reactivation from latency in vitro. Duplicated genes were targeted because of the increase in frequency of cleavage events as well as the potential to result in the division of the genome into two segments, each incapable of replicating if both cuts occurred in the same genome. This strategy eliminates the need for two separate vectors targeting unique region genes in order to generate dual-cleaved genomes, as performed in the study of Aubert et al., who showed that cleavage of the related HSV genome at two sites was more effective at reducing reactivation frequency than a single cleavage event [33]. Aubert et al. used two designer meganucleases to target different sites in HSV-1 that required simultaneous delivery by two different vectors and targeted duplicated genes in the repeated regions of the HSV genome. However, even with single cleavage events, error-prone DNA repair mechanisms that mutate the VZV ORF62 gene appear to reduce progeny viral replication due to the critical roles of IE62 in expression [34].

An important aspect of developing therapies from CRISPR/Cas enzymes is achieving an efficient delivery of the required gRNA and enzymes to the appropriate cell types. This is especially challenging for VZV, where the reservoir of latent viral genomes resides in ganglionic neurons throughout the peripheral nervous system. Therefore, the first steps in this project were to optimize the delivery of these molecules with a focus on human neurons. While both lentiviruses and AAV have been exploited for efficient gene delivery, current AAV vectors have the advantage in that they show little or no integration into the host genome [95]. However, the gene-packaging limits of AAV necessitate the use of smaller gene-editing enzymes, since the widely used Streptococcus pyogenes CRISPR/Cas9 is above the normal AAV packaging limit. Many smaller alternatives have recently been overviewed [96] and the Staphylococcus aureus CRISPR/Cas9 was chosen for its reported higher specificity, lower off-target activity in mouse neuroblastoma and liver cell lines [37], and the fact that it is within the packaging limits of AAV vectors [38]. Surveying four different AAV serotypes that have been shown to be neurotropic revealed that AAV2 efficiently delivered GFP to both hESC-derived neurons and two VZV-permissive epithelial cell lines. While we only tested four serotypes, it is possible that other serotypes could be found to improve transduction or decrease the number of AAV required. We do note, however, that AAV2 is one of the most commonly used and tested serotypes used in human studies and has been applied for gene delivery to repair multiple genetic diseases, some of which are now being evaluated in clinical trials [97,98].

Our studies show that delivering AAV-62-1gR-saCas9 decreased VZV progeny infectivity dramatically in both epithelial cells and hESC-derived neurons, compared in no AAV and no gRNA (“AAV-saCas9”) controls. This was shown by the reduction of both progeny-virus plaque size and numbers. Intriguingly, VZV that survived AAV-62-1gR-saCas9 treatment generated significantly smaller plaques when seeded onto naïve VZV-permissive cells, particularly VZV derived from AAV-targeted neurons. This strongly suggests that the virus produced in AAV-62-1gR-saCas9-treated cells is damaged, most likely as a result of genome cleavage and error-prone DNA repair, resulting in indels from the activation of the dsDNA damage response. Given that ORF62 is an essential gene whose expression is required for the expression of most other VZV genes, we postulate that some of the still-replicating but impaired viruses in these plaques are damaged but still have ORF62 in frame that permits expression of an ORF62 protein with some functional mutations. The site of the gRNA target in region 1 of ORF62/71 is that which the Cas9 acted upon, because VZV with mutations in that region (“VZV DR-62gmut”) showed only a minor loss of replication efficiency compared to the parental strain, but were completely resistant to pretreatment with AAV-62-1gR-saCas9. This establishes that the Cas9 did not influence VZV replication because of off-targeting of the host genome, although we cannot exclude that off-targeting effects might have also occurred that did not affect VZV replication.

One of the exciting potential applications of gene editing as an antiviral strategy is that it cannot only target productive replication of the virus, but can also damage the latent genomes and potentially prevent reactivation [89]. Treatment of latently infected and reactivated cultures of hESC derived neurons [23] with AAV-62-1gR-saCas9 resulted in greatly decreased spread of reactivation foci compared to controls. The number and size of foci that developed from reactivated neurons seeded onto ARPE-19 from AAV-62-1gR-saCas9-treated reactivated cultures also significantly decreased. Results from quantitative PCR measuring genome copies in the reactivated neurons revealed that treatment with AAV-62-1gR-saCas9 reduced replication but did not indicate a reduction of the genome load. Taken together, these results suggest that AAV-62-1gR-saCas9, in addition to reducing the viral load in lytic/productively infected cells, is an effective strategy to reduce replication in reactivated neurons. Of note, we have not yet been able to demonstrate whether latent genomes were cleaved by the targeting AAV-62-1gR-saCas9. We did not observe a loss of genome numbers in latently infected neurons after AAV-62-1gR-saCas9 treatment, but this may be a technical issue due to the very low levels of latent VZV DNA in neuronal cultures, which were insufficient to detect a significant change resulting from CRISPR/Cas9 treatment. A recent report found that gene editing of lytic HSV-1 is efficient but the editing of the chromatin-silenced latent genome required the expression of ICP0. Furthermore, ICP0 is known to alter protective chromatin, which may block the latent genome from being accessed by gene editors [83]. Similar strategies to target HSV-latent and lytic replicating genomes in an in vivo murine model of latency showed statistically significant but relatively minor reductions in the latent genome load, and also suggested relatively poor activities of CRISPR/Cas9 compared to meganuclease-mediated targeting [31,33,57,69,83]. A future possibility would be to evaluate the targeting of ORF62 by a designer meganuclease and to determine if it is more effective than the CRISPR/Cas9 strategy. However, we feel that a better direction would be to seek improvement in targeting by expressing multiple gRNAs to additional viral targets simultaneously with the single CRISPR/Cas9 nuclease.

The results presented here demonstrate the potential of the CRISPR/Cas9 strategy to reduce both epithelial manifestations of the reactivated disease and potential damage in the ganglia that could result from ganglionic spread after a reactivation event has initiated. Of course, much remains to be worked out in translating this approach to prevent zoster and VZV disease as a therapy. A possible initial application of AAV-mediated gene editing as an antiviral strategy could be for VZV-induced retinal diseases. The retina has been a prime target for advancing AAV-mediated gene therapy strategies, and there are promising results from both animal models and human studies that AAV-mediated delivery can restore vision loss for specific inherited defects [99]. VZV replication in the retinal tissues is known to result in rare but devastating blinding diseases such as peripheral outer retinal necrosis (PORN), acute retinal necrosis (ARN) and chorioretinitis [100]. It is not uncommon for these patients to respond poorly to classic antiviral therapies, because of delayed diagnoses and/or antiviral resistance [101]. We speculate that AAV-mediated antiviral gene editing in the eye following intravitreal delivery could be an alternative treatment strategy to prevent VZV-induced retinal damage. Future work in the treatment of zoster and other VZV diseases will require additional optimization for in vivo delivery as well as the early detection of triggered VZV reactivation events in vitro, neither of which have been well-addressed for CRISPR/Cas9 therapy for other herpesviruses to date. In addition, there is a need to overcome the host antiviral immune response to AAV vectors, since clinical trials have demonstrated that this can prevent the long-term use and expression of AAV-delivered genes [102]. There remains a need for antivirals to zoster, because some studies suggest that zoster is showing increased incidence longitudinally in populations under 50 that are not yet eligible for immunization with the zoster vaccines [103]. Nevertheless, these results serve as an important proof-of-principle of the CRISPR/Cas9 approach for treatment of a widespread and painful human disease for which there are currently few therapeutic options.

## Figures and Tables

**Table 1:** gRNA Oligos.

Gene	Direction	Primer Sequence (5′ → 3′)
**ORF62-1**	F	CACCGCTGGTTGAAGTCCCGATACGGA
	R	AAACTCCGTATCGGGACTTCAACCAGC
**ORF62-2**	F	CACCGCCGGCTTTTTACCCGAGATGGA
	R	AAACTCCATCTCGGGTAAAAAGCCGGC
**ORF62-3**	F	CACCGCAGCGCTCTACACCCCAACGCG
	R	AAACCGCGTTGGGGTGTAGAGCGCTGC
**ORF63-1**	F	CACCGATACGCGGGTGCAGAAACCG
	R	AAACCGGTTTCTGCACCCGCGTATC
**ORF63-2**	F	CACCGAAGACGGGTTCATTGAGGCG
	R	AAACCGCCTCAATGAACCCGTCTTC
**ORF63-3**	F	CACCGTTGAATTTCGGGATTCCGACG
	R	AAACCGTCGGAATCCCGAAATTCAAC
**UL3-4**	F	CACCGGTGACGAGCGCGATCCGGC
	R	AAACGCCGGATCGCGCTCGTCACC

**Table 2:** Primers for Quantification of AAVs.

Gene	Direction	Primer Sequence (5′ → 3′)
**SaCas9**	L	AGAAATACGTGGCCGAACTG
	R	TCACGTAGTCGCTGGTCTTG
**ORF49 (VZV genome)**	F	CGGTCGAGGAGGAATCTGTG
	R	CCGTTGCACGTAACAAGCTC

**Table 3:** Primer Design for VZV DR-62gmut BAC Recombineering.

Gene	Direction	
**62 gRNA region primers (5′ → 3′) ^a^**	F	GTCATGGTGGGACGGGAACATGAGATCGTTTCAATTCCCagtGTcagtGGcCTgCAgCCtGAACCCAGAAGGATG*ACTACGATAAGTAGG*
	R	TTGTGTTAGCTCTTCGCCAACATCTTCCGTTCTGGGTTCaGGcTGcAGgCCactgACactGGGAATTGAAA*GGGTAATGCCAGTGTTAC*
**Wobble base pair changes ^b^**	VZV DR	TCC GTA TCG GGA CTT CAA CCA G → S_68_ V_69_ S_70_ G_71_ L_72_ Q_73_ P_74_
	VZV DR-62gmut	agt GTc agt GGc CTg CAg CCt G → S_68_ V_69_ S_70_ G_71_ L_72_ Q_73_ P_74_

^a^ = 62 gRNA region primers that were used to alter the gRNA region. Uppercase non-italic letters denote bases that match sequence to that of VZV ORF62 pOka wild-type sequence; bases in italics denote sequence that recognizes the kanamycin resistance cassette for PCR amplification. Lowercase letters represent altered bases designed to change the 62-1 gRNA recognition site. ^b^ = Wobble base pair changes that were engineered into the gRNA recognition sequence are shown for VZV DR-62mut, while the parental wild-type sequence is shown for VZV DR. The top line in each segment shows the DNA sequence and the bottom line shows the encoded amino acids as one-letter amino acids and their residue numbers in the ORF62 protein. Lower case letters indicate the mutated bases engineered by the primers.

## Data Availability

The data in this study is available in this article, as well as upon reasonable request from the corresponding author.

## Supplementary Materials

The following supporting information can be downloaded at: https://www.mdpi.com/article/10.3390/v14020378/s1, Figure S1: Enlarged images from Figure 2b to show the GFP expression from different AAV serotypes after transduction into neuron cultures; Figure S2: Enlarged microscopy images from Figure 6a, showing that AAV-62-1gR-saCas9 treatment of latently infected neurons reduces VZV progeny and spread following reactivation induction.
