# Supplementary material for: Antiviral Targeting of Varicella Zoster Virus Replication and Neuronal Reactivation Using CRISPR/Cas9 Cleavage of the Duplicated Open Reading Frames 62/71
Source: Viruses. 2022 Feb 12;14(2):378. doi: 10.3390/v14020378 (PMC8880005; doi:10.3390/v14020378)
Supplement: Supplementary file 1 [file viruses-14-00378-s001.zip › viruses-1577348-supplementary.pdf]

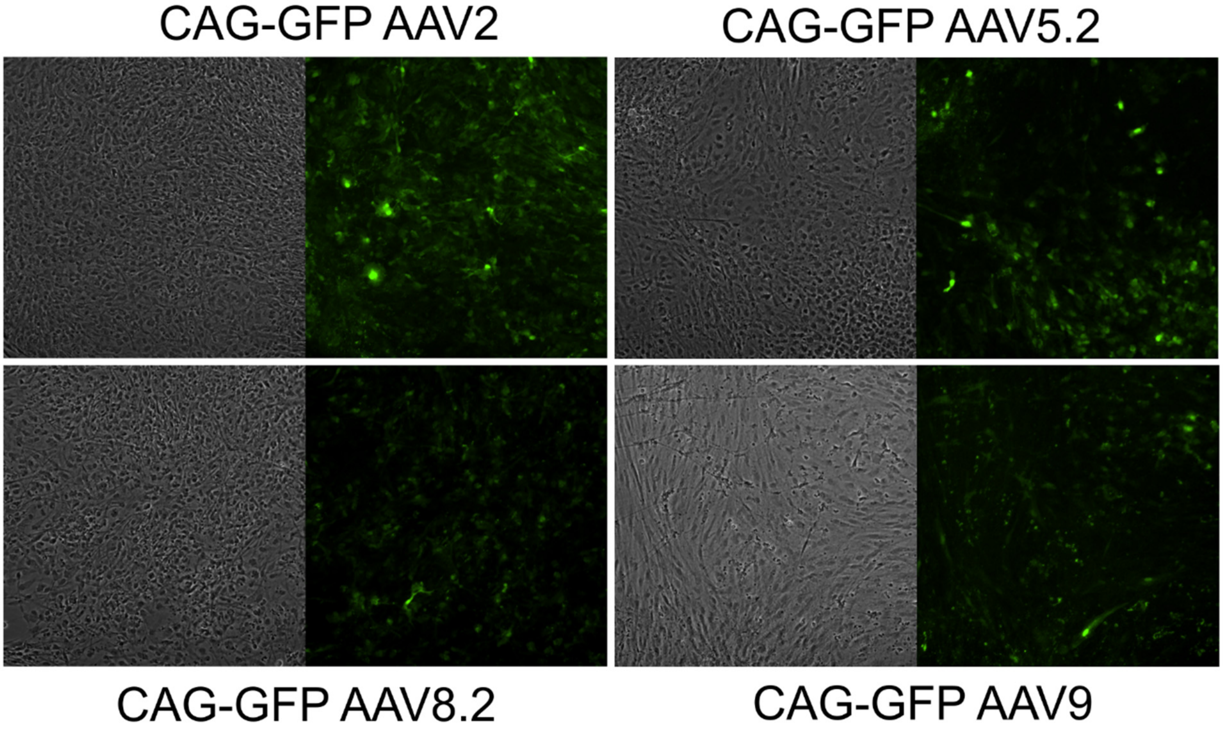

Figure S1. Enlarged images from figure 2b to show the GFP expression from different AAV serotypes after transduction into neuron cultures.

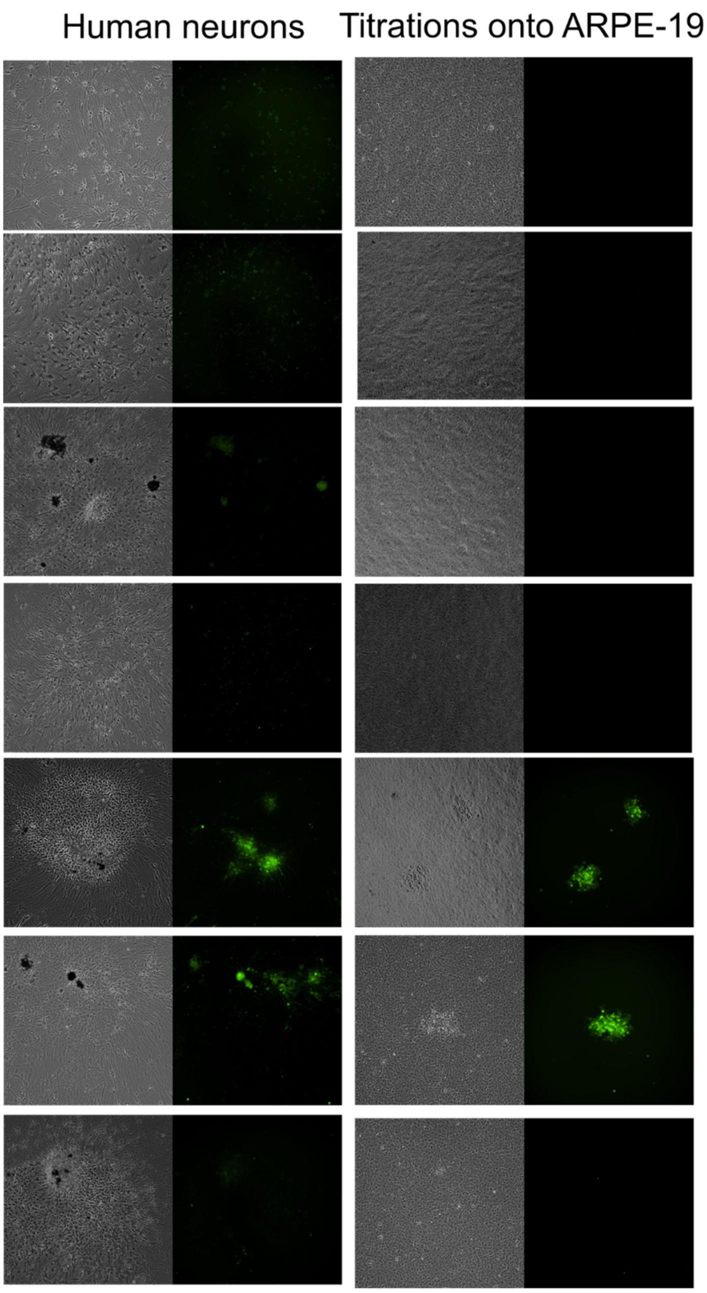

VZV treatment	AAV treatment
none	none
VZV66GFP no reactivation stimuli	none
none	AAV-saCas9 (no gRNA)
none	AAV-62-1gR-saCas9
VZV66GFP reactivated	none
VZV66GFP reactivated	AAV-saCas9
VZV66GFP reactivated	AAV-62-1gR-saCas9

Figure S2. Enlarged microscopy images from figure 6a, showing that AAV-62-1gR-saCas9 treatment of latently infected neurons reduces VZV progeny and spread following reactivation induction.
